# Pharmacokinetics of chewed vs. swallowed raltegravir in a patient with AIDS and MAI infection: some new conflicting data

**DOI:** 10.1186/s12981-014-0041-8

**Published:** 2015-01-17

**Authors:** Christoph D Spinner, Florian Wille, Christiane Schwerdtfeger, Philipp Thies, Ursula Tanase, Guido Von Figura, Roland M Schmid, Werner J Heinz, Hartwig Hf Klinker

**Affiliations:** Department of Medicine II, University Hospital Klinikum rechts der Isar, Ismaningerstr 22, 81675 Munich, Germany; Interdisciplinary HIV Centre (IZAR), University Hospital Klinikum rechts der Isar, Ismaningerstr 22, 81675 Munich, Germany; TUM Medical School, Technische Universitaet Muenchen, Ismaningerstr 22, 81675 Munich, Germany; Department of Medicine II, Division of Infectious Diseases, University Of Wuerzburg Medical Center, Oberduerrbacherstr 6, 97080 Wuerzburg, Germany

**Keywords:** HIV, Chewed, Raltegravir, Mycobacterium avium, Pharmacokinetic

## Abstract

**Background:**

While HIV, AIDS and atypical Mycobacterium infections are closely linked, the use of Integrase-Inhibitor based cART, notably raltegravir-based regimens is more widespread. RAL should be double-dosed to 800 mg semi-daily in situation of rifampicin co-medication, because RAL is more rapidly metabolized due to rifampicin-induced Uridine-5’-diphosph- gluronosyl-transferase (UGT1A1). Recently, it was speculated that chewed RAL might lead to increased absorption, which might compensate the inductive effect of rifampicin-rapid metabolized RAL, as part of cost-saving effects in countries with high-tuberculosis prevalence and less economic power.

**Methods:**

We report measurement of raltegravir pharmacokinetics in a 34-year AIDS-patient suffering from disseminated Mycobacterium avium infection with necessity of parenteral rifampicin treatment. RAL levels were measured with HPLC (internal standard: carbamazepine, LLQ 11 ng/ml, validation with Valistat 2.0 program (Arvecon, Germany)). For statistical analysis, a two-sided Wilcoxon signed rank test for paired samples was used.

**Results:**

High intra-personal variability in raltegravir serum levels was seen. Comparable C_max_ concentrations were found for 800 mg chewed and swallowed RAL, as well as for 400 mg chewed and swallowed RAL. While C_max_ seems to be more dependent from overall RAL dosing than from swallowed or chewed tablets, increased AUC_12_ is clearly linked to higher RAL dosages per administration. Anyway, chewed raltegravir showed a rapid decrease in serum levels.

**Conclusions:**

We found no evidence that chewed 400 mg semi-daily raltegravir in rifampicin co-medication leads to optimized pharmacokinetics. There is need for more data from randomized trials for further recommendations.

## Background

While Human Immunodeficiency Virus (HIV), Aquired Immunodeficiency Syndrome (AIDS) and atypical Mycobacteriosis infections are closely linked, the use of Integrase-Inhibitor (INI) based antiretroviral regiments (ART), notably raltegravir (RAL)-based regiments is more widespread during HAART era. Raltegravir’s benefit is the favorable drug-interaction profile whilst treating AIDS-defining events in HIV- patients [1]. Rifampicin is known to be active against atypical Mycobacterial infections, including Mycobacterium avium infections [2]. [3] Nevertheless, RAL is more rapidly metabolized in case of rifampicin co-administration due to rifampicin-induced Uridine-5’-diphosph-gluronosyl-transferase (UGT1A1) [1,4]. Therefore, it is recommended to double-dose RAL to 800 mg semi-daily (BID) in case of Rifampicin-use, which causes high additional costs in these patients [4]. Recently, there has been conflicting data, which suggests using a standard-dose of 400 mg RAL BID, taken chewed as part of an ART, might, in the event of Rifampicin co-administration, lead to higher drug absorption and lower drug intersubject pharmacokinetic variability [5,6]. As reported recently, it was speculated that chewed RAL might lead to increased absorption, which might thus compensate the inductive effect of rapid metabolized RAL due to rifampicin and therefore have cost-saving effects in countries with high-tuberculosis prevalence and less economic power [5]. Despite that aspect, some data for high inter- and intra-personal variability in pharmacokinetics were reported for HIV-patients in general [7]. Lack of sufficient RAL levels were not clearly associated with virological failure of antiretroviral therapy, but that there might be some influences of overall plasma exposure of the drug [7,8]. Therefore, conflicting data in differential RAL dosages play a major role in decisions on clinical HIV-patient care and overall antiretroviral therapy success.

### Clinical objectives

We report our experience with a recent admission of a 34-year-old Caucasian male HIV-patient, who was diagnosed as being HIV/HBV-co-infected 5 years prior to actual presentation. CDC Stage was C3, WHO stage IV due to thrush esophagitis and systemic cytomegalovirus reactivation. CD4-nadir = 8 cells/μl, he was known to belong to the risk group of men having sex with men.

4 months before current presentation at our center, the patient was diagnosed with mycobacterium avium infection in a lymph node biopsy whilst having fever, weight loss and general lymphadenopathy in a peripheral hospital. Antiretroviral therapy with co-formulated Tenofovir disoproxil fumarate and Emtricitabine (TVD) in combination with integrase-inhibitor RAL was initiated. Oral antimicrobial chemotherapy with rifabutin, ethambutol and clarithromycin was started without delay. The patient was discharged from the peripheral hospital. Subsequently, persistent fever and weight loss appeared within the first months.

The clinical situation worsened despite medical therapy. He was admitted to our emergency department with shock and severe hypoglycemia 4 months after initiation of the first antimycobacterial treatment. The marasmic patient (body mass index of 15), suffered from 5 liters of watery diarrhea per day, severe hypoalbuminemia (albumin 1,4 g/dl), protein deficiency (serum protein 4,4 g/dl), and iron deficiency anemia (hemoglobin 7,0 g/dl). Subsequently, mycobacterium avium was diagnosed by microscopy and culture in samples of pleural effusion, stool, sigmoid biopsy and peripheral blood. No other microorganisms could be identified in blood culture diagnostics. Due to the lack of intestinal absorption, antimicrobial chemotherapy was escalated to parenteral rifampicin (600 mg once daily), ethambutol (400 mg three times daily), clarithromycin (500 mg semi-daily) and amikacin (750 mg once daily).

Before adjusting ART to the recommended dose of 800 mg RAL BID (adult formulation) in combination with Tenfovir disoproxil fumarate co-formulated with Emtricitabine, subsequent pharmacokinetic studies were performed after informed consent of the patient. In doing so, RAL concentrations were measured in serum before, 1, 2, 4, 8 and 12 hours after a BID oral application of RAL with a dosage of 400 mg and 800 mg. Before new measurement after every dose adjustment, a lead-in phase of more than 24 hours was performed. For optimized reliability, chewed RAL dosage studies were performed semi-daily at different points of time. At any time, a standard meal was provided and pantoprazole was co-administered at a dosage of 40 mg once daily. Usage of pH-altering agents like pantoprazole was associated with a possible increase in AUC and C_max_ in recent studies [9]. However, the clinical impact of pH-altering agents and RAL levels was less important in HIV/AIDS patients, compared to healthy volunteers [10].

## Methods

RAL levels were measured with high performance liquid chromatography (HPLC, internal standard: carbamazepine, LLQ 11 ng/ml, validation with Valistat 2.0 program (Arvecon, Germany)). For statistical analysis, a two-sided Wilcoxon signed rank test for paired samples was used.

## Results

**Figure 1:**
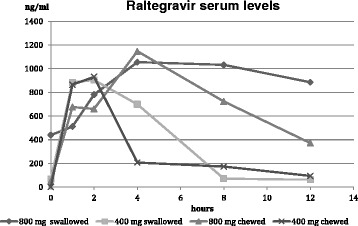
**Pharmacokinetic data of raltegravir after administration of different dosages (400 mg or 800 mg, BD; either chewed or swallowed).**

Overall, comparable RAL C_2h_ concentrations were found with either chewed or swallowed 400 mg or 800 mg of RAL BID in the case of rifampicin co-administration. Similarly C_max_ concentrations were found for 800 mg chewed and swallowed RAL BID, as well as for 400 mg chewed and swallowed RAL BID. While C_max_ seems to be more dependent on overall RAL dosing than on swallowed or chewed tablets, increased AUC_12_ is clearly linked to higher RAL dosages per administration. Chewed or swallowed RAL in a dosage of 800 mg BID led to acceptable AUC_12_ levels (8767 ng/ml; 10965 ng/ml), whereas 400 mg RAL BID showed significantly lower AUC_12_ levels when chewed or swallowed (3783 ng/ml; 4778 ng/ml). Interestingly, lower AUC_12_ levels were seen in chewed dosages of RAL, compared to a similar dose of swallowed RAL in 400 mg and 800 mg. For chewed or swallowed 400 mg RAL dosages, a rapid decrease in serum levels was seen, beginning 2–4 hours after application. These findings were confirmed in low serum levels at the beginning of the next medication at C_0_ or C_12_. Our finding results in dangerously low serum concentrations 8–12 hours after application of chewed or swallowed 400 mg RAL BID, as well as in low C_0_ concentrations (<45 ng/ml; 66 ng/ml). Notably lower C_0_ RAL levels were also seen for the chewed 800 mg RAL BID dosage when compared to the equivalent chewed 800 mg RAL BID dosage (47 ng/ml). Although a relevant intra-personal variability in raltegravir serum levels in this HIV-patient with watery diarrhea and intestinal mal-absorption due to systemic Mycobacterium avium infection, have to be taken into account, only swallowed 800 mg RAL led to a lasting RAL serum concentration of 439 ng/ml at C_0_ and 885 ng/ml at C_12_ with acceptable AUC_12_ of 10965 ng/ml. Pharmacokinetic data in detail are shown in Table 1, a graph of RAL pharmacokinetics is shown in Figure 1. However, a comparison of RAL concentrations after chewed and swallowed administration of RAL showed no statistically significance.

**Table 1:** **Pharmacokinetic data of Raltegravir after administration of different dosages of raltegravir by chewing or swallowing the drug in a patient with AIDS and MAI infection**

**Parameter**	**Raltegravir concentration (ng/ml)**			
	**400 mg** ***swallowed***	**400 mg** ***chewed***	**800 mg** ***swallowed***	**800 mg** ***chewed***
C_0h_	66	<45	439	47
C_1 h_	884	865	511	676
C_2 h_	904	930	781	660
C_4 h_	699	207	1055	1147
C_8 h_	71	173	1032	723
C_12 h_	62	94	885	372
AUC_0–4_	2972	2489	2957	2837
AUC_12_	4778	3783	10965	8767
***Difference AUC***_***12***_ *(ng/ml); median; min; max*	−3,5;-102;492		215;-165;513	
***(p-value)***	*(p-value 0,84)*		*(p-value 0,22)*

## Conclusion

Based on these findings we could not demonstrate optimized RAL levels after taking chewed compared to swallowed RAL tablets in contrast to previously reported data [5]. Even though there might be changes in RAL pharmacokinetics due to pantoprazole co-administration in our data, we would expect a 3–4 fold higher C_max_ and AUC of RAL levels. It can be speculated that the RAL levels might be less high as here reported in the absence of gastric proton pump inhibitors. This might be another risk factor for lower RAL levels in case of rifampicin co-administration. Anyhow, whilst potential cost-saving effects of RAL dosing savings may be important, the risk of virological failure due to resistance-evolution plays a crucial role in INI-based antiretroviral therapy. Therefore, we keep using double-dosed swallowed RAL tablets in the case of rifampicin co-medicated patients, as recommended by the official prescribing information, until further data from controlled trials are available. Dose reduction can cause relevant and potentially dangerous decreases in RAL serum levels and AUC_12_, which can cause serious harm to HIV-patients. Potential cost-saving effects should not endanger effective antiretroviral therapy, until there is further evidence of any other superior strategy.
